# Establishing and applying an adaptive framework for imported malaria: a field practice in Anhui Province, China from 2012 to 2022

**DOI:** 10.1186/s12889-024-18239-w

**Published:** 2024-03-04

**Authors:** Tao Zhang, Xian Xu, Bowen Liu, Duoquan Wang, Xiangguang Ye, Jingjing Jiang, Shuqi Wang, Xiaofeng Lyu, Chen Yu, Cuicui Tian, Zijian Liu, Xuechun Lu, Shizhu Li, Weidong Li

**Affiliations:** 1 Anhui Provincial Center for Disease Control and Prevention, 12560 Fanhua Road, 230601 Anhui, Hefei China; 2 School of Global Health, Chinese Centre for Tropical Diseases Research, Shanghai Jiao Tong University School of Medicine, 200025 Shanghai, China; 3 National Key Laboratory of Intelligent Tracking and Forecasting for Infectious Diseases, National Institute of Parasitic Diseases at Chinese Center for Disease Control and Prevention, Chinese Center for Tropical Diseases Research; NHC Key Laboratory of Parasite and Vector Biology; WHO Collaborating Center for Tropical Diseases, National Center for International Research on Tropical Diseases, 200025 Shanghai, China; 4 Anhui Intermational Travel Healthcare Center, 230002 Hefei, China

**Keywords:** Imported malaria, Prevention of re-establishment of malaria, Strategy, Framework, Anhui Province, China

## Abstract

**Background:**

Anhui Province is currently facing an increase in imported malaria cases as a result of globalization and international travel. In response, Anhui Province has implemented a comprehensive adaptive framework to effectively address this threat.

**Methods:**

This study collected surveillance data from 2012 to 2022 in Anhui Province. Descriptive statistics were used to analyze the epidemiological characteristics of imported malaria cases. Additionally, multivariate logistic regression was employed to identify factors associated with severe malaria. Documents were reviewed to document the evolution of the adaptive framework designed to combat imported malaria. The effectiveness of the adaptive framework was evaluated based on the rates of timely medical visits, timely diagnosis, and species identification.

**Results:**

During the study period, a total of 1008 imported malaria cases were reported across 77 out of 105 counties in Anhui Province, representing a coverage of 73.33%. It was found that 10.52% of imported cases went undiagnosed for more than seven days after onset. The multivariate analysis revealed several potential risk factors for severe malaria, including increasing age (OR = 1.049, 95%CI:1.015–1.083), occupation (waitperson vs. worker, OR = 2.698, 95%CI:1.054–6.906), a longer time interval between onset and the initial medical visit (OR = 1.061, 95%CI:1.011–1.114), and misdiagnosis during the first medical visit (OR = 5.167, 95%CI:2.535–10.533). Following the implementation of the adaptive framework, the rates of timely medical visits, timely diagnosis, and species identification reached 100.00%, 78.57%, and 100.00%, respectively.

**Conclusions:**

Anhui Province has successfully developed and implemented an adaptive framework for addressing imported malaria, focusing on robust surveillance, prompt diagnosis, and standardized treatment. The experiences gained from this initiative can serve as a valuable reference for other non-endemic areas.

## Introduction

Malaria, a disease transmitted by mosquitoes and caused by protozoan parasites, continues to be a significant global public health issue. Progress has been made in reducing the malaria burden worldwide over the past twenty years. In 2021, there were 84 malaria-endemic countries compared to 108 in 2000 [1]. However, the coronavirus disease 2019 (COVID-19) pandemic disrupted health services and led to an increase in malaria cases. It is estimated that there were 247 million malaria cases worldwide in 2021, resulting in approximately 619,000 deaths [1]. During this challenging period, countries certified by the World Health Organization (WHO) as malaria-free, including China, must make continuous efforts to prevent the re-establishment of malaria and support global progress towards elimination.

In China’s history, malaria has been a significant health concern. In the 1940s, there were at least 30 million cases of malaria annually with a fatality rate of about 1% [2]. After the establishment of the People’s Republic of China, the national malaria control program implemented adaptive strategies and integrated interventions, leading to a significant reduction in the malaria burden over the following decades [3, 4]. The National Malaria Elimination Action Plan (2010–2020), launched in 2010 [5], aimed to eliminate malaria nationwide by 2020. Guided by this action plan, local transmission was interrupted, and the last indigenous cases were reported in Yunnan province in 2016 [6]. On June 30, 2021, China was certified as malaria-free by the WHO, marking a new milestone in global malaria elimination [7].

Anhui Province, located in southeastern China, initiated a provincial malaria elimination plan in 2010. No locally transmitted cases have been reported since 2014. By 2019, Anhui Province had maintained its malaria-free status for six years and received sub-national malaria elimination certification. In May 2021, the province, representing China due to its high receptivity to malaria, underwent evaluation by an independent Malaria Elimination Certification Panel established by the WHO. However, the province still faces imported malaria cases due to globalization and increased international travel [8, 9]. Preventing the re-establishment of malaria is now a top priority in the province, and an adaptive framework has been gradually established and implemented to address this concern. This paper presents an adaptive framework based on epidemiological evidence, showcasing practices and experiences in developing innovative interventions for preventing the re-establishment of malaria. The adaptive framework is a flexible and dynamic system that primarily focuses on interdepartmental cooperation, health education for at-risk populations, surveillance, and case management to address imported malaria. Its implementation will contribute to the advancement of global malaria elimination efforts.

## Methods

### Study design

A retrospective case-based study was conducted using surveillance data to analyze imported malaria in Anhui Province from January 2012 to December 2022. In addition, government documents, regulations, departmental cooperation agreements, and technical protocols related to imported malaria were collected and reviewed from 2014 onward to examine the evolution of an adaptive framework. The framework was specifically designed to address the threat of imported malaria, and these materials provided valuable insights into its development. To evaluate the effectiveness of the adaptive framework, indicators such as rates of timely medical visits, timely diagnosis, and species identification were utilized. The study aimed to assess the impact of the adaptive framework across three dimensions: timeliness of treatment, timeliness of diagnosis, and the ability to identify the species of malaria parasite.

### Malaria case

#### Confirmed case

It is defined as a malaria case or infection in which the presence of the parasite has been detected through a diagnostic test [10]. In Anhui Province, blood samples were collected from malaria cases by county-level CDC staff prior to administering antimalarial treatment. These samples were then sent to the Anhui Provincial Malaria Diagnostic Reference Laboratory, which was established in 2012. The laboratory conducted tests using microscopic examination of Giemsa-stained blood films and real-time Polymerase Chain Reaction (PCR) analysis. Commercialized kits provided by Shanghai ZJ Bio-tech Co., Ltd. were utilized to differentiate between different species of malaria parasites. Each PCR reaction consisted of a 35 µL mixture, 0.4 µL Taq enzyme, 1 µL internal control, and 4 µL DNA template. Negative and positive controls were included to monitor the reactions. The reaction conditions were set according to the instructions provided with the kits: 37℃ for 2 min followed by 94℃ for 2 min, followed by 40 cycles at 93℃ for 15 s and 60℃ for 60 s.

#### Imported case

It is defined that an imported malaria case or infection refers to a situation where the individual acquired the disease outside the geographical area (China) where it is diagnosed [10].

#### Severe case

Severe malaria was determined based on the clinical features and laboratory test results in accordance with the “Diagnosis of Malaria” health standard (WS 259–2015) issued by the National Health and Family Planning Commission of China [11]. More specifically, it refers to confirmed cases exhibiting one or more of the following symptoms: loss of consciousness or coma, severe anemia, renal impairment, pulmonary edema, or Acute Respiratory Distress Syndrome.

### Data collection

In China, malaria is categorized as a notifiable infectious disease [12]. Any suspected cases must be reported to the Centers for Disease Control and Prevention (CDC) through the China Information System for Disease Control and Prevention (CISDCP). Once a report is received, professionals from the county-level CDC carry out an epidemiological investigation utilizing a standardized questionnaire. The collected information is then entered into the Information System for Parasitic Disease Control and Prevention (ISPDCP), which is a subsystem of the CISDCP. Additionally, an epidemiological report is generated to document the valuable insights obtained during the investigation.

For this study, demographic, epidemiological, and clinical data of malaria cases were extracted from the CISDCP, ISPDCP, and epidemiological survey reports. Since 2018, the Anhui Provincial CDC and Anhui Provincial Center for Clinical Laboratories have jointly conducted an annual external competency assessment to evaluate the diagnostic capabilities of general hospitals in Anhui Province regarding malaria microscopy. This assessment involves the random examination of five blood slides per hospital using optical microscopy, with each slide scrutinized for the presence of malaria parasites. If a positive blood slide is identified, species identification is performed. The assessment data was included in the study.

Furthermore, in this paper, we have collected and reviewed documents relating to the prevention and control of imported malaria in Anhui Province since 2014. The aim is to outline the functions and operational mode of the adaptive framework.

### Statistical analysis

Descriptive and comparative statistics were utilized to portray the epidemiological profile of imported malaria. Normally distributed continuous variables were displayed as mean and standard deviation (SD), whereas non-normally distributed variables were exhibited as median and interquartile range (IQR). Categorical data were summarized in terms of numbers and percentages. Differences in proportions were evaluated using appropriate statistical tests such as Pearson’s chi-squared χ2 or Fisher’s exact test. The chi-squared test for linear trend was utilized to determine whether there was a significant time trend. Logistic regression models were employed to identify the connection between risk factors and severe malaria, and the outcomes were depicted using odds ratios (ORs) and 95% confidence intervals (CIs). The Box-Tidwell test was carried out to assess the assumption of linearity between the logit of the outcome and the continuous variable. If the assumption was not met, the continuous variable was changed into a categorical variable. The dummy variable method was employed to deal with missing categorical data. The statistical significance level was set as *P* < 0.05. SPSS version 26.0 (SPSS Inc., Chicago, IL, USA) was used for all statistical tests. MapInfo 15.0 (Pitney Bowes, Troy, NY) was utilized to create the thematic map.

## Results

### Epidemiological profile

**Fig. 1:**
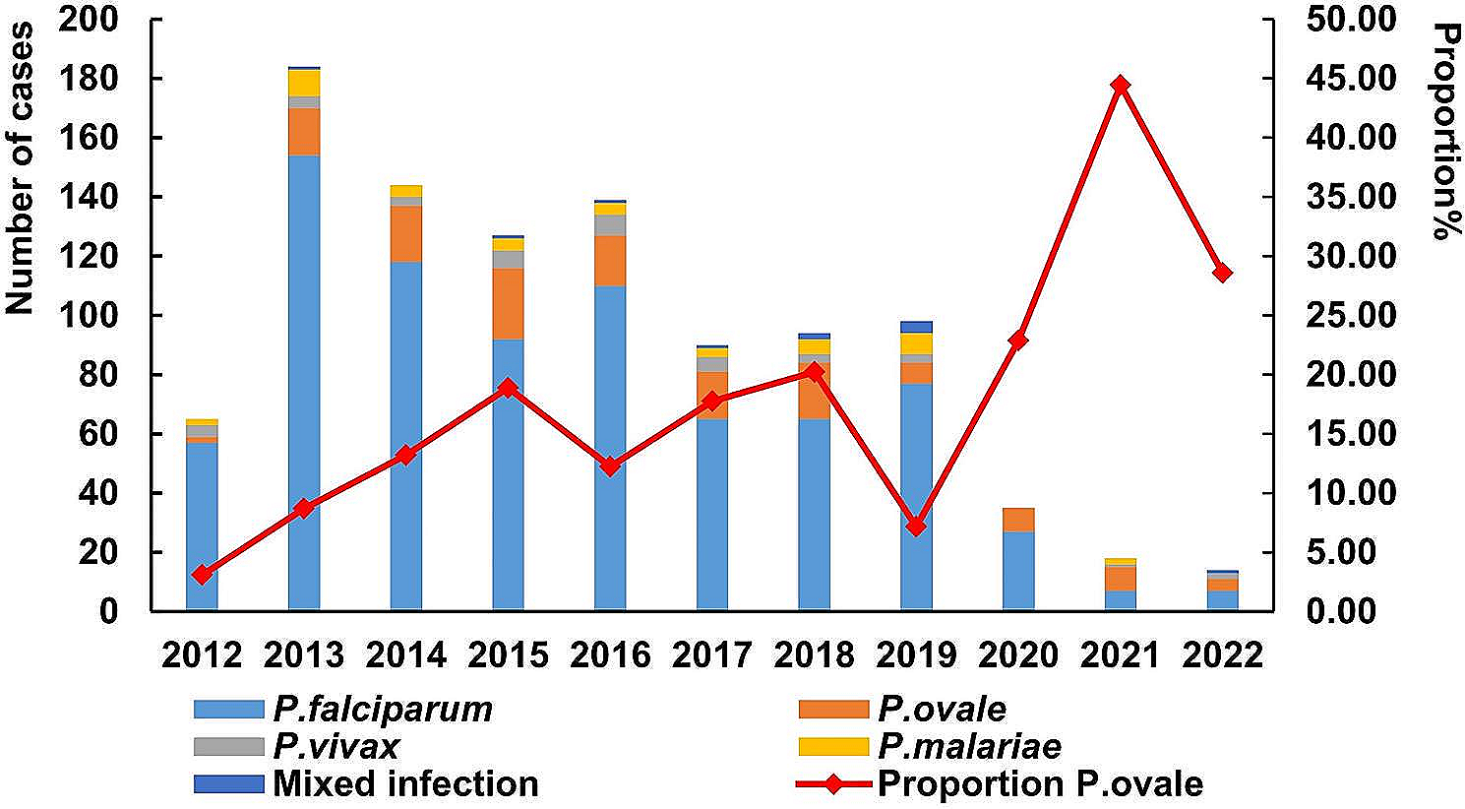
Imported malaria in Anhui Province, 2012–2022

Throughout the study, a total of 1008 imported malaria cases were reported in Anhui Province, with annual cases ranging from 14 to 184. In 2013, the highest number of cases was recorded at 184, accounting for 18.25% of the total cases. However, during the COVID-19 pandemic (Fig. 1), only 67 cases were reported between 2020 and 2022. Among the 1008 infections, *Plasmodium falciparum* (*P. falciparum*) was the most common species, accounting for 779 cases (77.28%). This was followed by* P. ovale* with 140 cases (13.89%), *P. malariae* with 40 cases (3.97%), *P. vivax* with 38 cases (3.77%), and mixed infections with 11 cases (1.09%). The percentage of *P. ovale* cases showed an increasing trend (χ2 = 13.690, *P* < 0.001), peaking in 2021 at 44.44% (Fig. 1).

The average age of reported cases was 41.28 ± 9.33 years, and 98.31% (991 cases) of the cases were male. The majority of cases (97.12%, 972/1008) were among overseas laborers, including workers and waitpersons. The infections originated from 32 African countries (992 cases, 98.41%) and 6 Asian countries (16 cases, 1.59%). Angola had the highest number of cases (238 cases, 23.61%), followed by Equatorial Guinea (144 cases, 14.29%), and Nigeria (130 cases, 12.90%). Imported malaria cases were reported in 73.33% (77/105) of the counties in the province during the study period. The top three counties with the highest number of cases were Feidong County with 300 cases (29.76%), Yaohai District with 73 cases (7.24%), and Shushan District with 61 cases (6.05%).

Severe malaria was reported in 59 patients (5.85%), resulting in six deaths and a mortality rate of 0.60%.

### The patient’s experience from symptom onset to diagnosis

**Fig. 2:**
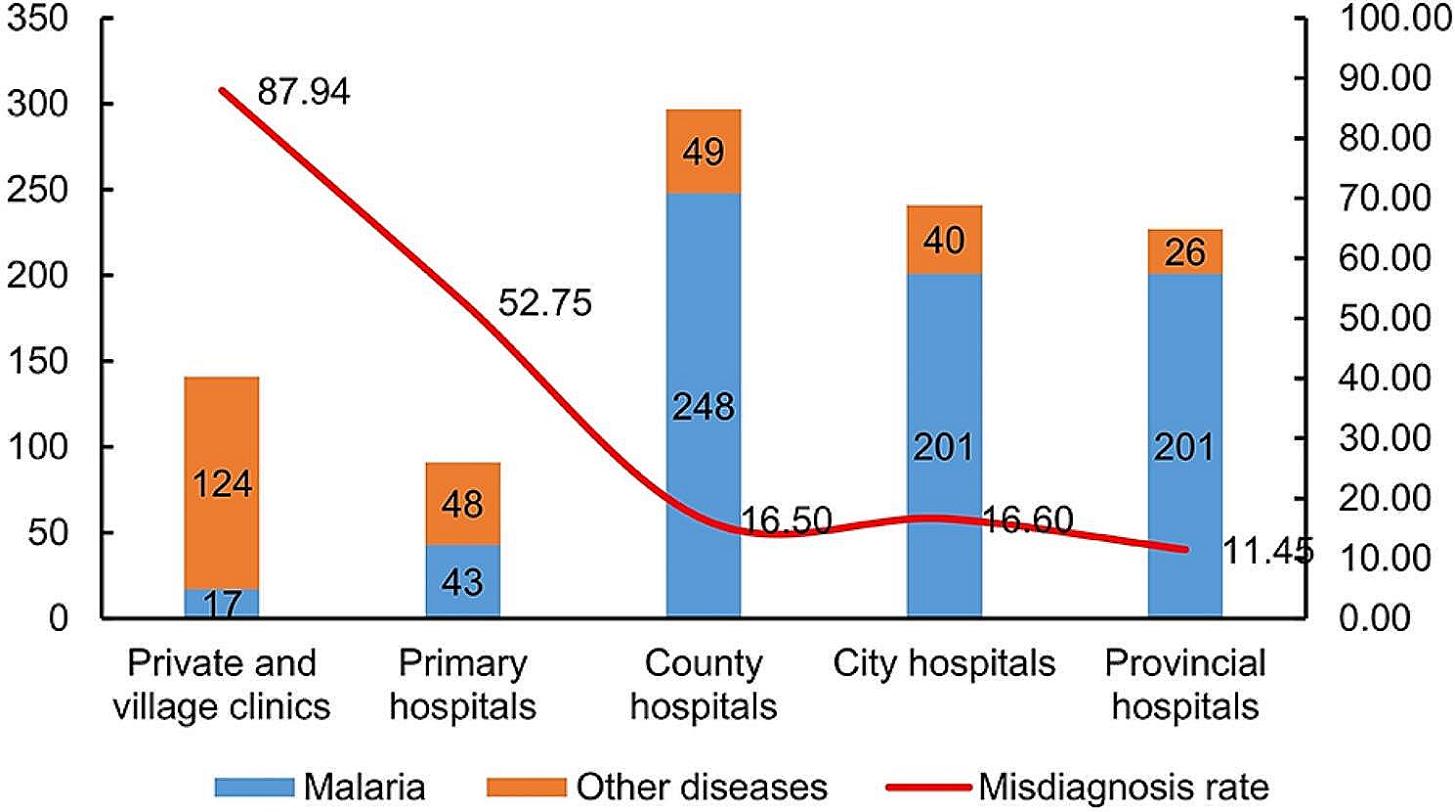
Diagnostic results of the first medical visit by the levels of medical facilities in Anhui Province from 2012 to 2022

During the first medical visit, 297 (29.46%) patients sought medical care in county-level hospitals, followed by 241 (23.91%) in city-level hospitals, 227 (22.52%) in provincial hospitals, 141 (13.99%) in private/village clinics, and 91 (9.03%) in primary hospitals. The remaining eleven patients sought medical services at other health institutions, such as overseas hospitals, the International Travel Health Care Center, and others. Notably, at the first health visit, the misdiagnosis rate in private and village clinics was up to 87.94% (124/141), and that in primary hospitals was 52.75% (48/91) (Fig. 2).

Of all cases, the median time from onset to the first medical visit, the first medical visit to diagnosis, and onset to diagnosis was 1 (IQR: 0–2 days) day, 1 (IQR: 0–2 days) day, and 2 (IQR: 1–5 days) days, respectively. Moreover, in some instances, patients were diagnosed more than seven days after onset, representing 10.52% of cases (Table 1).

**Table 1:** Time between symptom onset and diagnosis of imported malaria cases in Anhui Province, 2012–2021

Time interval	Days, median (IQR)	≤ 3 days		4–7 days		>7 days	
		N	%	N	%	N	%
Time from onset to first medical visit	1(0,2)	853	84.62	120	11.90	35	3.47
Time from first medical visit to diagnosis	1(0,2)	855	84.82	111	11.01	42	4.17
Time from onset to diagnosis	2(1,5)	652	64.68	250	24.80	106	10.52

### Influence factors for severe malaria

Potential factors influencing severe malaria were identified using logistic regression analysis. The results of the univariate analysis indicated that sex, duration between symptom onset and first medical visit, time from the first medical visit to diagnosis, level of health facility for initial medical visit, diagnosis outcome of the first medical visit, and history of previous malaria infection were significantly associated with severe malaria. Variables with a P-value < 0.10 were included in the multivariable model.

The multivariable model revealed that older age (OR = 1.049, 95%CI: 1.015–1.083), occupation as a waiter/waitress compared to a worker (OR = 2.698, 95%CI: 1.054–6.906), a prolonged interval between symptom onset and initial medical visit (OR = 1.061, 95%CI: 1.011–1.114), and misdiagnosis during the first medical visit (OR = 5.167, 95%CI: 2.535–10.533) were identified as risk factors for severe malaria. In contrast, male patients (OR = 0.110, 95%CI: 0.027–0.442) and patients with a history of previous malaria infection (OR = 0.392, 95%CI: 0.212–0.727) had significantly lower chances of developing severe malaria (Table 2).

**Table 2:** Logistic analysis of factors associated with the risk of severe malaria

Factor	The number of cases, N(%)	Odds ratio (95% CI)			
		Crude	P-value	Adjusted	P-value
**Sex**					
Female	17(1.69)	**reference**		**reference**	
Male	991(98.31)	0.138(0.047–0.407)	< 0.001	0.110(0.027–0.442)	0.002
**Age (years),mean(SD)**	41.28(9.33)	1.027(0.998–1.058)	0.070	1.049(1.015–1.083)	0.004
**Occupation**					
Worker	902(89.48)	**reference**		**reference**	
Waiter	77(7.64)	2.109(0.958–4.641)	0.064	2.698(1.054–6.906)	0.038
Other	29(2.88)	2.911(0.973–8.705)	0.056	1.746(0.459–6.649)	0.414
**Duration of stay overseas (days)**					
≤ 30	34(3.37)	**reference**		**reference**	
31–180	180(17.86)	0.885(0.281–2.786)	0.835	3.208(0.828–12.427)	0.092
181–365	237(23.51)	0.330(0.097–1.120)	0.075	1.150(0.280–4.733)	0.846
> 365	525(52.08)	0.328(0.106–1.013)	0.053	1.252(0.329–4.770)	0.742
Missing	32(3.17)	NA	NA	NA	NA
**Time from onset to visit (days), median (IQR)**	1(0,2)	1.052(1.007–1.098)	0.022	1.061(1.011–1.114)	0.016
**Time from visit to diagnosis (days), median (IQR)**	1(0,2)	1.084(1.032–1.139)	0.001	1.014(0.946–1.088)	0.690
**The level of health facilities for initial medical visit**					
Private and village clinics	141(13.99)	**reference**		**reference**	
Primary hospitals	97(9.62)	0.651(0.257–1.650)	0.366	0.966(0.350–2.671)	0.947
County-level hopsitals	297(29.46)	0.300(0.136–0.666)	0.003	0.769(0.299–1.979)	0.586
City-level hospitals	241(23.91)	0.482(0.228–1.020)	0.056	1.193(0.481–2.957)	0.703
Provincial hospitals	227(22.52)	0.398(0.179–0.884)	0.024	1.283(0.488–3.372)	0.613
Other	11(1.09)	NA	NA	NA	NA
**The diagnosis result of first medical visit**					
Malaria	716(71.03)	**reference**		**reference**	
Other diseases	292(28.97)	4.951(2.851–8.599)	< 0.001	5.167(2.535–10.533)	< 0.001
**Previous malaria**					
No	385(38.19)	**reference**		**reference**	
Yes	623(61.81)	0.271(0.155–0.476)	< 0.001	0.392(0.212–0.727)	0.003

### Blind examination of malaria blood slides

During the study period, five rounds of external competence assessments were conducted. The hospitals consistently maintained a qualitative accuracy of over 90% each year, except for 2018, which showed a statistically significant upward trend. Similarly, the rate of species identification exceeded 60% each year, with a peak of 70.11% in 2022 (Table 3).

**Table 3:** Results of blinded examination of malaria blood slides in Anhui Province, 2012–2022

Year	The number of hospitals	Qualitative accuracy ^a^(%)	Accuracy of species identification^b^(%)
2018	110	89.45(492/550)	57.11(217/380)
2019	129	93.33(602/645)	63.22(275/435)
2020	129^c^	92.86(585/630)	62.82(267/425)
2021	129^c^	95.56(602/630)	63.68(270/424)
2022	129	95.66(617/645)	70.11(305/435)
χ^2^		2161.697	117.081
*P-value*		< 0.001	< 0.001
Trend χ^2^		182.588	0.006
*P-value*		< 0.001	0.938

^a^The proportion of blood slides that can be accurately determined whether protozoa were present

^b^For positive slides, the proportion of blood slides that can be correctly identified species

^c^Three hospitals in 2020 and three in 2021 did not report their assessment results

### An adaptive framework for imported malaria

**Fig. 3:**
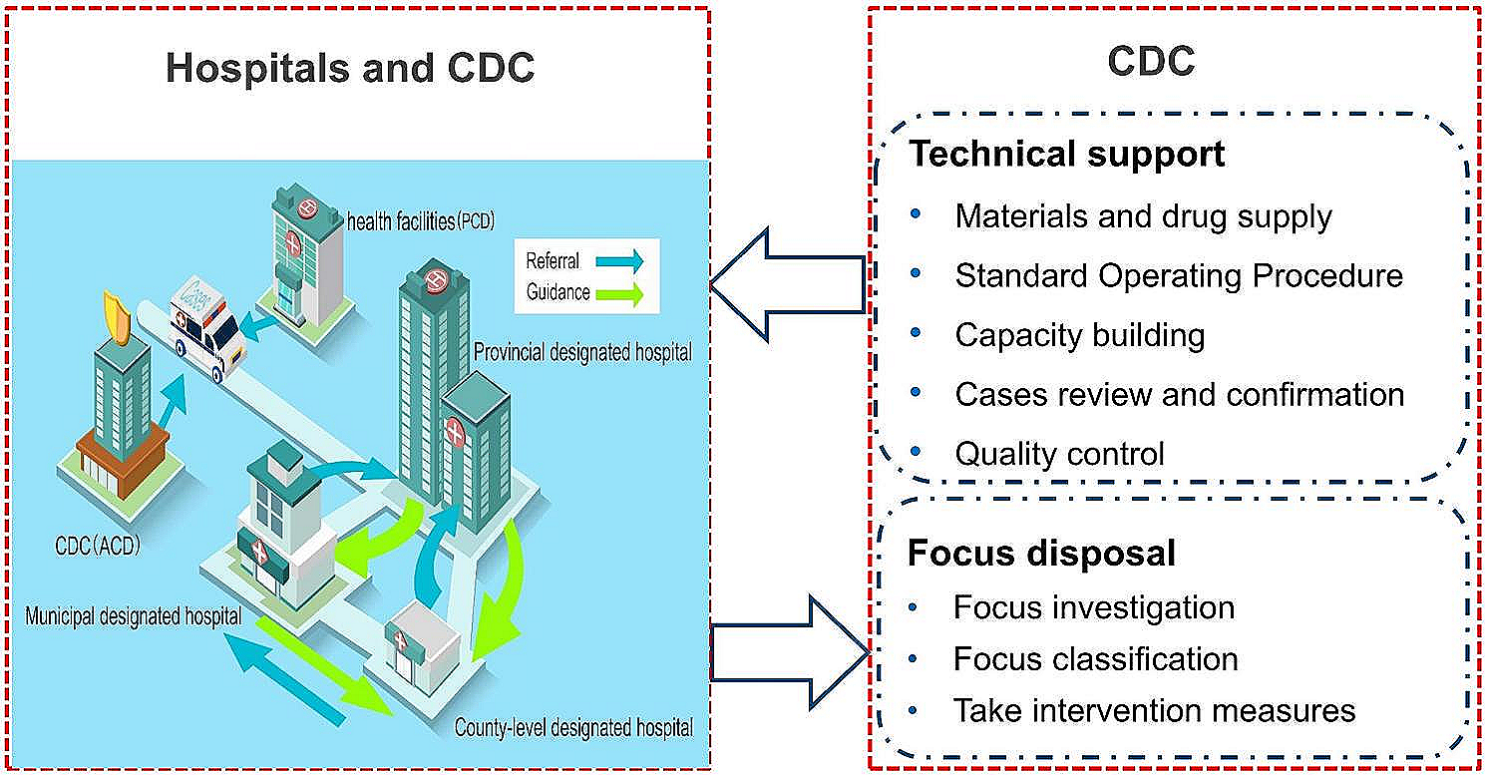
The surveillance and response system for imported malaria in Anhui Province

**Fig. 4:**
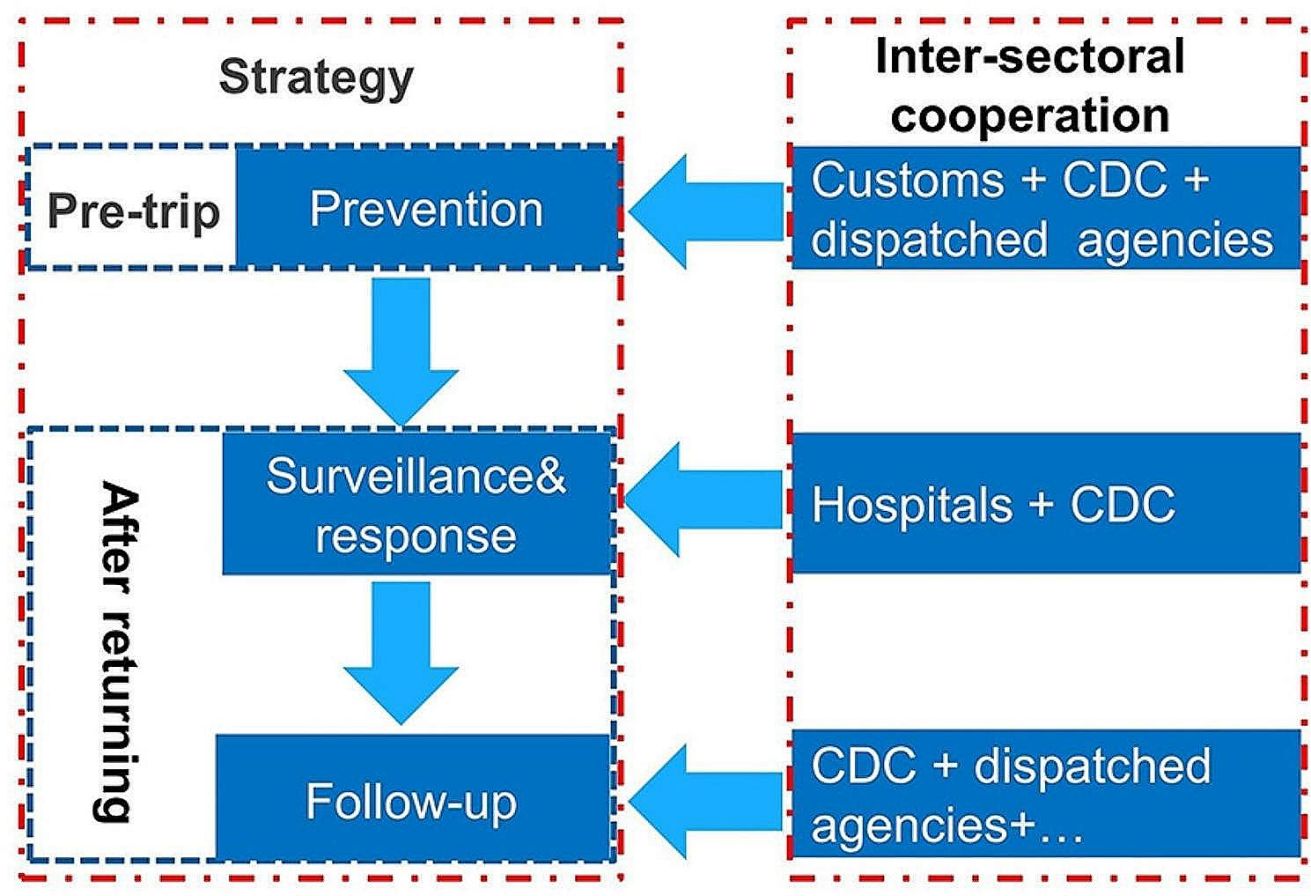
Joint prevention and control mechanisms for imported malaria between departments in Anhui Province

An adaptive framework for managing imported malaria has been progressively established in Anhui Province. The framework is based on four key elements: governmental leadership, surveillance and response, intersectoral cooperation, and capacity building. First and foremost, the government has provided a supportive environment, including policies, funds, and workforce guarantees. Within this context, a surveillance network for malaria covering the entire province (Fig. 3) has been established and sustained, which uses passive case detection (PCD) and active case detection (ACD). PCD primarily involves microscopy or rapid diagnostic tests (RDTs) performed on suspected malaria cases in health facilities. ACD refers to the detection of malaria infections in populations considered to be at high risk and is usually conducted by CDC staff. Once the surveillance system detects malaria patients, they are transferred to designated hospitals for medical observation and appropriate treatment. Presently, there are 104 designated hospitals in Anhui Province treating malaria cases, including 82 county-level hospitals, 18 city-level hospitals, and four provincial-level hospitals that cater to severe malaria patients. Moreover, these hospitals store antimalarial drugs and offer professional staff training in their respective regions. Meanwhile, the CDC provides technical assistance to designated hospitals to improve case detection and management, including provisions of malaria test materials and antimalarial drugs, Standard Operating Procedures (SOP) for microscopy and RDTs, capacity building for staff (such as training of doctors and inspectors, blind examination of malaria blood slides), case confirmation, and quality control for laboratory tests. For all malaria cases, the CDC provides feedback within three days of case confirmation to assist health providers in developing treatment plans and taking prompt measures following the “1-3-7” strategy [13] to minimize the risk of malaria re-establishment. Anhui Province has been exploring collaborative prevention and control mechanisms among different departments, as shown in (Fig. 4). Initially, the Anhui CDC and Hefei Customs, in collaboration with dispatched agencies, provided interventions (such as health education and mosquito repellent) for at-risk populations before they departed from China. Secondly, the health administration ensures collaboration among hospitals and the CDC to promptly and precisely identify malaria infections and to provide appropriate treatment. Finally, the CDC and dispatch agencies monitor malaria cases to understand their prognosis.

### The preliminary effect of the adaptive framework

**Fig. 5:**
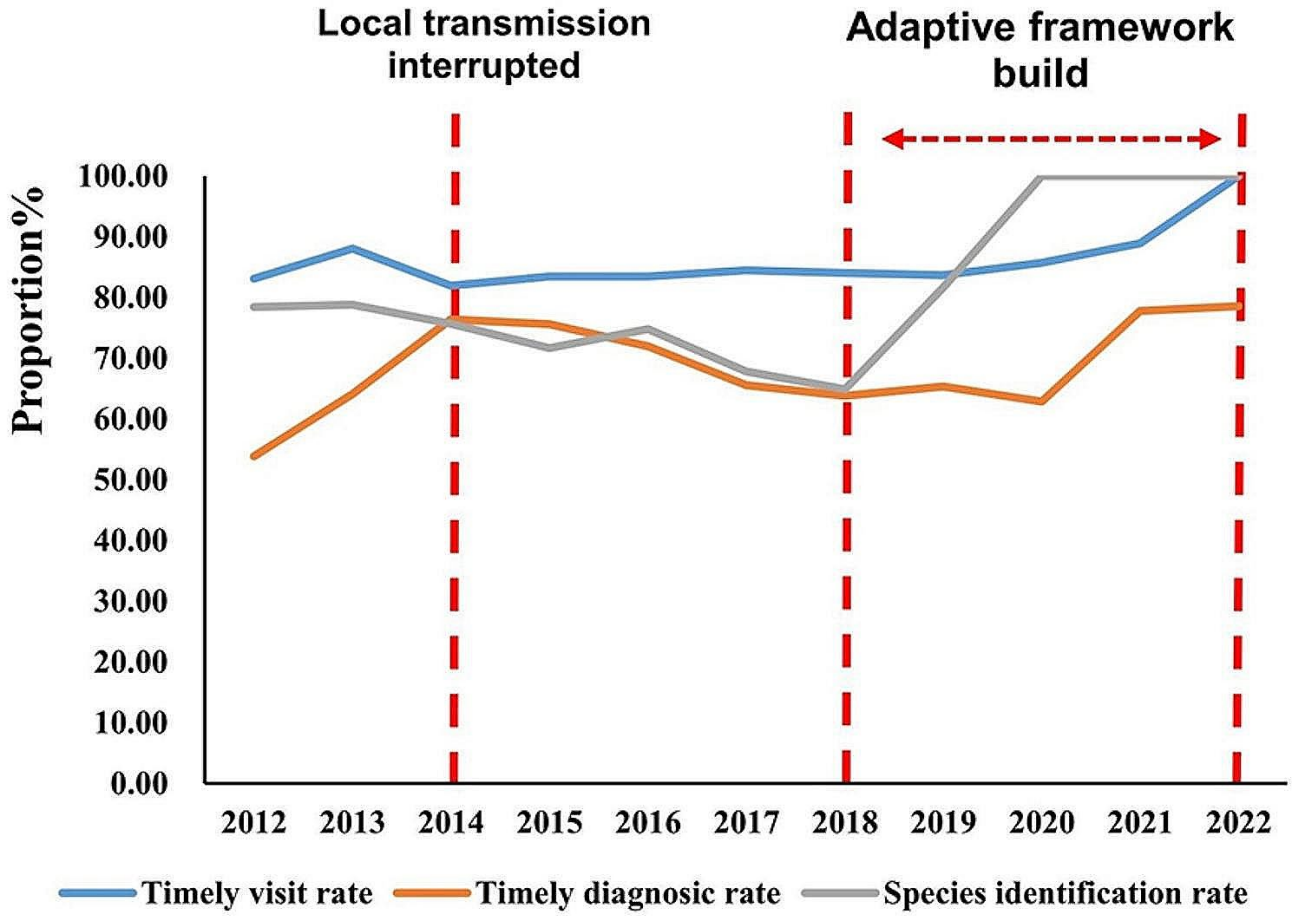
Effect of the adaptive framework for malaria in Anhui Province

This study defines a timely visit within the first 72 h of onset, as identified in previous studies [14, 15]. Additionally, a diagnosis made within the first 24 h after the visit is also considered timely. The rate of species identification is calculated based on the number of correctly identified species among malaria cases. After the interruption of local transmission, rates of timely diagnosis and species identification declined, while timely medical visits remained stable, as depicted in Fig. 5. In particular, timely diagnosis decreased from 76.39% in 2014 to 63.83% in 2018, and species identification decreased from 75.69% in 2014 to 64.89% in 2018. However, the implementation of the adaptive framework in 2018 led to an increase in rates for timely medical visits, timely diagnosis, and species identification. By 2022, the rates reached 100.00%, 78.57%, and 100.00% for timely medical visits, timely diagnosis, and species identification, respectively (Fig. 5).

## Discussion

Anhui Province was one of the provinces most severely affected by malaria in China. Local transmission of malaria in Anhui Province has been interrupted since 2014. Nevertheless, with globalization and increased international travel, imported malaria has become a challenge for the province.

Imported malaria cases, different from local transmission, are widely distributed throughout Anhui Province and occur throughout the year. Over the study period, 73.33% of counties in the province reported imported malaria cases, but in small numbers. In fact, healthcare providers in Anhui Province, particularly at the primary level, lack essential awareness and skills to manage imported malaria due to a lack of experience. Therefore, health facilities’ ability to remain vigilant against imported malaria cases is challenging. The study found that 10.52% of imported cases remained undiagnosed for more than seven days after the initial onset. Additionally, misdiagnosis at the first medical visit increases the risk of severe malaria. These results are consistent with prior studies that have shown delayed diagnosis among patients with imported malaria is common in non-endemic areas, further leading to severe malaria [16–18]. Delayed care-seeking is another concern. Existing literature has reported that patients with imported malaria do not seek healthcare promptly even after symptom onset [19], reflecting a lack of awareness of malaria among patients. Sometimes, they tend to self-treat symptoms [20]. Notably, our study results indicate that a longer interval between the onset of symptoms and the initial medical visit increases the likelihood of severe malaria. Additionally, low species identification rates also adversely affect case management due to treatment protocols specific for different species [21]. Five rounds of external competence assessment have been conducted in Anhui Province since 2018. The rate of species identification fluctuated between 57.11% and 70.11% for the assessed hospitals, which is insufficient for standard treatment.

The strategies and measures adopted in the POR phase need to be tailored to meet the aforementioned challenges. Therefore, an adaptive framework for imported malaria has been progressively established in Anhui Province, which involves prevention, surveillance, and treatment of imported malaria. In a supportive environment created by the government, multiple relevant sectors work together to tackle imported infectious diseases. First, the epidemiological profile of imported malaria in Anhui is consistent with previous provincial and national studies [22–25], except for the China-Myanmar border region [26]. Patients are mainly migrant laborers returning from Africa, with the majority being middle-aged males [9]. The Anhui CDC, along with Hefei Customs and labor dispatch agencies, initially collaborated to offer targeted interventions to the population at higher risk. These interventions primarily consist of providing health education and mosquito repellent. In comparison to Western nations, where malaria is often acquired by citizens after a brief visit to their country of origin [27, 28], approximately half of our labor force remains outside China for more than a year [29]. The extended duration of overseas stays makes chemoprophylaxis difficult, as its efficacy relies on regular administration, which may be challenging due to poor compliance and limited availability. Furthermore, manufacturers have discontinued the production of these medications due to the domestic market contraction. Therefore, despite its proven effectiveness based on present evidence [30], the accessibility to chemoprophylaxis in China is currently poor.

Meanwhile, a malaria surveillance and treatment network has been established in the province by the CDC and hospitals to promptly detect and manage cases of malaria. Monitoring activities include both PCD and ACD. PCD is primarily performed in health facilities. Rapid diagnostic tests (RDTs) were introduced to enhance the sensitivity of PCD in Anhui Province, in combination with microscopy. It is an effective strategy as it possibly detects more P. falciparum infections [31]. ACD refers to the CDC carrying out health follow-up and screening for malaria upon receiving information about returnees from endemic areas. In addition, the CDC is responsible for organizing capacity-building activities such as annual training sessions for doctors and inspectors. Since 2018, the Anhui CDC and Anhui Provincial Center for Clinical Laboratories have jointly conducted an external yearly quality assessment through a blind examination of malaria blood slides. Additionally, skill competitions were held, and winners were rewarded. The objective of these programs is to enhance the capability of diagnosing and treating imported malaria at health facilities. Health-care providers are required to report suspected malaria cases to the local CDC within 24 h of detection through a web-based health information system. Upon receiving the report, the CDC takes prompt measures according to the “1-3-7” work strategy [13] to reduce the risk of local transmission.

For the treatment of imported malaria cases, 104 designated hospitals have been established to cover the whole province. These hospitals maintain a stock of antimalarial medication and provide personnel training on appropriate treatment methods for malaria. This training ensures that staff have the necessary skills to effectively care for individuals suffering from malaria. Meanwhile, by stocking antimalarial drugs, the hospitals are able to provide prompt treatment to patients. During the study period, *P. falciparum* accounted for nearly 80% of registered malaria cases, which can develop severe complications and lead to rapid death [32]. To minimize the impact of severe malaria, Anhui Province has designated four provincial level hospitals as specialized centers for intensive care and has assembled a group of experts in intensive care for malaria. From 2012 to 2022, a total of six deaths were reported, with a mortality rate of 0.60%, which is close to the level of the USA [33]. For all malaria cases, the CDC provides feedback on the results of case confirmation to help health providers develop treatment plans and conducts follow-up assessments to determine their prognosis with the support of labor dispatch agencies as necessary.

The adaptive framework was developed between 2018 and 2022, building upon previous work. The implementation of the adaptive framework resulted in increased rates of timely medical visits, diagnoses, and species identification. However, due to strict COVID-19 prevention measures during the implementation of the framework, the observed effect cannot solely be attributed to the framework. Therefore, long-term observation and evaluation are necessary to accurately measure the impact of the adaptive framework.

## Conclusions

Robust surveillance, prompt diagnosis, and standardized treatment are imperative for POR in the post-elimination phase. In response, Anhui Province has developed an initial adaptive framework to meet these goals. The field experience gained from this endeavour will serve as a reference for other non-endemic areas.

## Data Availability

The datasets analyzed during the current study are available in a Figshare repository: 10.6084/m9.figshare.23945688.

## Abbreviations

ACD: active case detection
CDC: Centers for Disease Control and Prevention
COVID-19: coronavirus disease 2019
CIs: confidence intervals
CISDCP: China Information System for Disease Control and Prevention
ORs: odds ratios
POR: prevent the re-establishment of malaria
ISPDCP: Information System for Parasitic Disease Control and Prevention
IQR: interquartile range
*P. falciparum*: *Plasmodium falciparum*
*P. malariae*: *Plasmodium malariae*
*P. ovale*: *Plasmodium ovale*
*P. vivax*: *Plasmodium vivax*
PCD: passive case detection
RDTs: rapid diagnostic tests
SD: standard deviation
WHO: World Health Organization

## References

[1] World Health Organization. World Malaria Report 2021. 2022. https://apps.who.int/iris/rest/bitstreams/1398397/retrieve. Accessed 2 July 2023.

[2] Feng J, Zhang L, Xia ZG, Zhou SS, Xiao N (2021). Malaria-free certification in China: achievements and lessons learned from the National Malaria Elimination Programme. Zoonoses.

[3] Tang L (2000). Progress in malaria control in China. Chin Med J (Engl).

[4] [Malaria situation in the People’s Republic of China in 1999] (2000). Zhongguo Ji Sheng Chong Xue Yu Ji Sheng Chong Bing Za Zhi.

[5] Ministry of Health of the People’s Republic of China. Action plan of China malaria elimination (2010–2020).2010. http://www.gov.cn/zwgk/ 2010-05/ 26/ conte nt_ 16141 76. htm. Accessed 2 July 2023.

[6] Feng J, Zhang L, Huang F, Yin JH, Tu H, Xia ZG, Zhou SS, Xiao N, Zhou XN (2018). Ready for malaria elimination: zero indigenous case reported in the people’s Republic of China. Malar J.

[7] Zhou XN. China declared malaria-free: a milestone in the world malaria eradication and Chinese public health. Infect Dis Poverty 2021, 10(1). 10.1186/s40249-021-00882-9 PMC8276478 34253259

[8] Zhang T, Xu X, Jiang J, Yu C, Tian C, Xie Q, Li W (2019). Risk factors of severe imported malaria in Anhui province, China. Acta Trop.

[9] Li W, Zhang T, Xu X, Jiang J, Yu C, Tian C, Wang S, Lyu X, Liu Z (2020). Problems Associated with the diagnosis of Imported Malaria in Anhui Province, China. Am J Trop Med Hyg.

[10] World Health Organization. WHO Malaria Terminology. 2016. https://www.who.int/publications/i/item/ 97892 40038 400. Accessed 2 July 2023.

[11] National Health and Family Planning Commission of China. Diagnosis of Malaria 2015. http://www.nhfpc.gov.cn/ewebeditor/uploadfile/2015/11/20151125105511210.pdf. Accessed 2 July 2023.

[12] Wang L, Wang Y, Jin S, Wu Z, Chin DP, Koplan JP, Wilson ME (2008). Emergence and control of infectious diseases in China. Lancet.

[13] Cao J, Sturrock HJ, Cotter C, Zhou S, Zhou H, Liu Y, Tang L, Gosling RD, Feachem RG, Gao Q (2014). Communicating and monitoring surveillance and response activities for malaria elimination: China’s 1-3-7 strategy. PLoS Med.

[14] Zhang T, Wang DQ, Qian YJ, Ruan W, Liu Y, Xia J, Yan H, Sui Y, Lu SN, Xu X et al. Profile and determinants of delayed care-seeking and diagnosis among patients with imported malaria: a retrospective study in China, 2014–2021. *Infect Dis Poverty* 2022, 11(1). 10.1186/s40249-022-01050-3 PMC9773583 36550586

[15] Lu GY, Cao YY, Chen Q, Zhu GD, Muller O, Cao J. Care-seeking delay of imported malaria to China: implications for improving post-travel healthcare for migrant workers. J Travel Med 2022, 29(4). 10.1093/jtm/taab156 PMC9282091 34581417

[16] Newman RD, Parise ME, Barber AM, Steketee RW (2004). Malaria-related deaths among US travelers, 1963–2001. Ann Intern Med.

[17] Daily JP, Minuti A, Khan N (2022). Diagnosis, treatment, and Prevention of Malaria in the US A Review. Jama-J Am Med Assoc.

[18] Bastaki H, Carter J, Marston L, Cassell J, Rait G (2018). Time delays in the diagnosis and treatment of malaria in non-endemic countries: a systematic review. Travel Med Infect Di.

[19] Lu GY, Cao YY, Chai LY, Li YP, Li SY, Heuschen AK, Chen Q, Muller O, Cao J, Zhu GD (2022). Barriers to seeking health care among returning travellers with malaria: a systematic review. Trop Med Int Health.

[20] Zhang M, Liu ZY, He HT, Luo L, Wang SQ, Bu HL, Zhou X (2011). Knowledge, attitudes, and practices on Malaria Prevention among Chinese International travelers. J Travel Med.

[21] National Health and Family Planning Commission of the People’s Republic of China. Technical regulations for application of antimalarials. 2016. http://www.moh.gov.cn/ewebeditor/uploadfile/2016/05/201605301434293-28.pdf. Accessed 2 July 2023.

[22] Yu T, Fu Y, Kong X, Liu X, Yan G, Wang Y (2020). Epidemiological characteristics of imported malaria in Shandong Province, China, from 2012 to 2017. Sci Rep.

[23] Lin K, Wei H, Jiang W, Li J, Zhang W, Wei S, Yang Y, Huang Y, Feng X, Tu H (2017). Malaria in the Guangxi Zhuang Autonomous Region in China: a twelve-year Surveillance Data Study. Am J Trop Med Hyg.

[24] Zhang X, Yao L, Sun J, Pan J, Chen H, Zhang L, Ruan W (2018). Malaria in Southeastern China from 2012 to 2016: analysis of Imported cases. Am J Trop Med Hyg.

[25] Feng J, Zhang L, Tu H, Zhou SS, Xia ZG (2021). From elimination to post-elimination: characteristics, challenges and re-transmission preventing strategy of imported malaria in China. Chin Trop Med.

[26] Xu JW, Lin ZR, Zhou YW, Lee RG, Shen HM, Sun XD, Chen QY, Duan KX, Tian P, Ding CL et al. Intensive surveillance, rapid response and border collaboration for malaria elimination: China Yunnan’s ‘’3 + 1’’strategy. Malar J 2021, 20(1). 10.1186/s12936-021-03931-8 PMC8502357 34627264

[27] Jelinek T, TropNetEurop. Imported Falciparum malaria in Europe: 2007 data from TropNetEurop. Euro Surveill 2008, 13(23). 18761952

[28] Cullen KA, Arguin PM, Division of Parasitic D, Malaria, CfGHCfDC. Prevention: Malaria surveillance–United States, 2011. *MMWR Surveill Summ* 2013, 62(5):1–17. 24172939

[29] Zhang T, Wang S, Wang D, Auburn S, Lu S, Xu X, Jiang J, Lyu X, Yu C, Tian C (2021). Epidemiological profile of Plasmodium ovale spp. imported from Africa to Anhui Province, China, 2012–2019. Malar J.

[30] Delaigue S, Signolet I, Consigny PH, de Gentile L, D’Ortenzio E, Gautret P, Sorge F, Strady C, Bouchaud O (2020). New guidelines for the prevention of imported malaria in France. Med Mal Infect.

[31] Li W, Zhang X, Feng J, Zhang T, Xu X, Jiang J, Wang S, Lyu X, Li S, Lu M (2021). Evaluation of the combination of rapid diagnostic tests and microscopy for imported malaria surveillance in Anhui Province, China. Acta Trop.

[32] Zhang L, Tu H, Zhou S, Xia Z, Feng J (2021). Malaria deaths - China, 2011–2020. China CDC Wkly.

[33] Mace KE, Lucchi NW, Tan KR. Malaria Surveillance - United States, 2018. *MMWR Surveill Summ* 2022, 71(8):1–35. 10.15585/mmwr.ss7108a1 PMC9470224 36048717

